# How a life-like system emerges from a simple particle motion law

**DOI:** 10.1038/srep37969

**Published:** 2016-11-30

**Authors:** Thomas Schmickl, Martin Stefanec, Karl Crailsheim

**Affiliations:** 1 Department for Zoology, Karl-Franzens University Graz, Austria

## Abstract

Self-structuring patterns can be observed all over the universe, from galaxies to molecules to living matter, yet their emergence is waiting for full understanding. We discovered a simple motion law for moving and interacting self-propelled particles leading to a self-structuring, self-reproducing and self-sustaining life-like system. The patterns emerging within this system resemble patterns found in living organisms. The emergent cells we found show a distinct life cycle and even create their own ecosystem from scratch. These structures grow and reproduce on their own, show self-driven behavior and interact with each other. Here we analyze the macroscopic properties of the emerging ecology, as well as the microscopic properties of the mechanism that leads to it. Basic properties of the emerging structures (size distributions, longevity) are analyzed as well as their resilience against sensor or actuation noise. Finally, we explore parameter space for potential other candidates of life. The generality and simplicity of the motion law provokes the thought that one fundamental rule, described by one simple equation yields various structures in nature: it may work on different time- and size scales, ranging from the self-structuring universe, to emergence of living beings, down to the emergent subatomic formation of matter.

The emergence of order from chaos is one of the key phenomena in morphogenesis across spatiotemporal scales^1^. Key singularities, in which order emerges and self-sustains from chaotic substrates, still wait for full understanding^2^: Our universe is self-structuring, self-replicating life-forms emerged from simple molecules and simple chemical interaction patterns produce persistent complex thoughts in our brains^3^,^4^,^5^,^6^. These structures reside at equilibrium of growth and decay, in which unordered particles are self-ordering and - after some time - fall back to unorderedness^1^,^7^, as living systems manage to reside far from a thermodynamic equilibrium^8^. No general simple proximate model yields such dynamic complex structures, and it is undecided whether there was “gene first” or “metabolism (cell) first”^9^,^10^ at the origin of life. We discovered a simple motion law for self-propelled particles (SPP)^11^ allowing spontaneous emergence of self-sustaining and self-reproducing protocells^12^ from populations of homogeneous reactive particles. These structures show distinct lifecycles with morphogenesis and reproduction. Thus we consider those structures to be protocells^12^ as they encapsulate the basic properties of very early life forms in the primordial soup^13^,^14^. We named this class of systems “Primordial Particle Systems” (PPS), as they model a spontaneous emergence of life-like structures. In contrast to existing protocell models^12^,^15^ PPS are significantly simpler without impairing their dynamics, thus they are more general and fundamental. Unlike other simple models, most prominently Conway’s “Game of Life”^16^, PPS produce natural-like structures (cells, spores) and dynamics (emergent ecology, emergent life cycles) without any restriction to unnatural modeling constraints like spatial discretization or time synchronization of agents. Thus, we claim that our PPS represents the simplest model capable of predicting the emergence of ordered structures that behave, interact, grow, heal, replicate, and die without showing high sensitivity on parameters (particle connectivity, speed, density), as we show by our results.

We aim for an as-simple-as-possible system that builds a life-like ecosystem from (random) scratch that is self-organizing in an emergent way based solely on local interaction (forces) without any global information available to the involved components. These restrictions make the system interesting for designing artificial “active matter” systems^17^: The simpler the microscopic rules are, and the more localized interaction is, and the more resilient and pluripotent the resulting emergent macroscopic system is against error/noise, the higher will be the system’s potential to produce interesting, maybe even life-like, artificial systems^18^.

Besides that, the generality and simplicity of the motion law provokes the thought that such structures observed on multiple size scales (from cosmic to sub-atomic) may emerge due to one fundamental rule described by a simple equation, like the one we present here.

## Method

### The model

A PPS models a population of particles moving deterministically and asynchronously in a continuous toroidal wrapped space. Each particle is defined by its position **p**_*t*_ = (*x*_*t*_*, y*_*t*_), by its heading *ϕ*_*t*_, and by its movement with constant velocity *v*, assuming an open system allowing steady energy influx for motion. Particles react to other particles within radius *r* by changing their heading: Turn directionality depends on particle numbers left (*L*_*t,r*_) and right (*R*_*t,r*_) of them. Turn angles depend on the total number of surrounding particles (*N*_*t,r*_ = *L*_*t,r*_ *+* *R*_*t,r*_). This yields the final motion law for PPS:


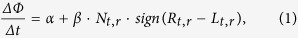


where a positive change of *ϕ* is a turn to the right. The parameter *α* represents a fixed rotation, *β* models a rotation proportional to local neighborhood size. Neighborhood configuration affects *ϕ*_*t*_ in each time step, in turn changing a particle’s position, ultimately yielding new local configurations. This feedback loop governs the self-organization of the PPS system.

With *α* = *180*°, isolated particles hold position within 2 time steps. Only when other particles enter their neighborhood they start to move away: PPS with *α* = *180*° ‘mirror’ the behavior of particles in PPS with *α* = *0*° on the microscopic level.

**Figure 1:**
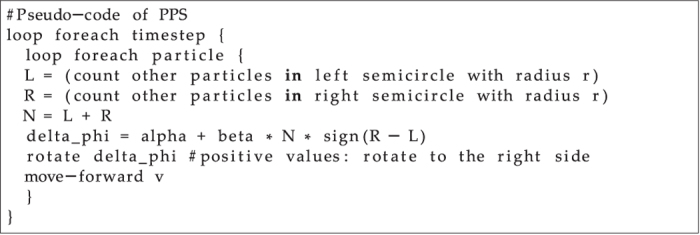
Explanatory implementation of a PPS as pseudo-code. Each particle determines the number of its neighbors on the left side (*L*_*t*_, all other particles in a semicircle with radius *r*) and on the right side (*R*_*t*_, all other particles in a semicircle with radius *r*). The change of its heading (*Δϕ*) in each time step *t* is the left-hand side result of equation 1. After rotating, each particle moves forward with a fixed velocity (*v*).

In our PPS each particle holds position **p**_*t*_ = (*x*_*t*_, *y*_*t*_) and heading *ϕ*_*t*_ at every time step t. The change of this heading (*Δϕ*) is modeled in our model equation 1. Every positive heading change is considered to be a turn to the right (first person view), every negative *Δϕ* is a left turn (first person view), respectively counter clockwise (third person view). The positional update is modeled according to **p**_t+1_ = **p**_t_ + ((*cos ϕ*_*t*_), (*sin ϕ*_*t*_)) ∙ *v*, where *v* is the constant velocity of each particle. Figure 1 shows a pseudo-code describing the algorithmic implementation.

We discovered an exceptionally interesting system with the parameter set *PPS* = 〈*r* = *5*, *α* = *180*°, *β* = *17*°, *v* = *0.67*〉, used as default PPS parameters here. Cell-like structures emerge spontaneously with around 0.08 particles/space unit (p/su) initially randomly distributed in a space of 250 × 250 units. For visualization we color-code particles by their local neighborhood size: *color*_*t*_ = *if 15* < *N*_*t,r=5*_ ≤ *35 blue else if N*_*t,r=5*_ > *35 yellow else if 13* ≤ *N*_*t,r=5*_ ≤ *15 brown else if N*_*t,r=1.3*_ > *15 magenta else green*.

The movement of particles was calculated in discrete time steps in continuous space (64 bit floating point positions and orientations of particles). Initially all particles were randomly distributed and randomly oriented (both uniform random distributions). All particles were moving in a randomized order within every time step of acting. They act asynchronously by our model of sensing and moving, which is performed within the same algorithmic loop, particle by particle. Local densities were indicated by particle colors following this set of rules based on local neighborhood within a specific radius: *color*_*t*_ = *if 15* < *N*_*t,r=5*_ ≤ *35 blue else if N*_*t,r=5*_ > *35 yellow else if 13* ≤ *N*_*t,r=5*_ ≤ *15 brown else if N*_*t,r=1.3*_ > *15 magenta else green*. The subscripts *t* denotes the time step and *r* denotes the radius.

**Figure 2:**
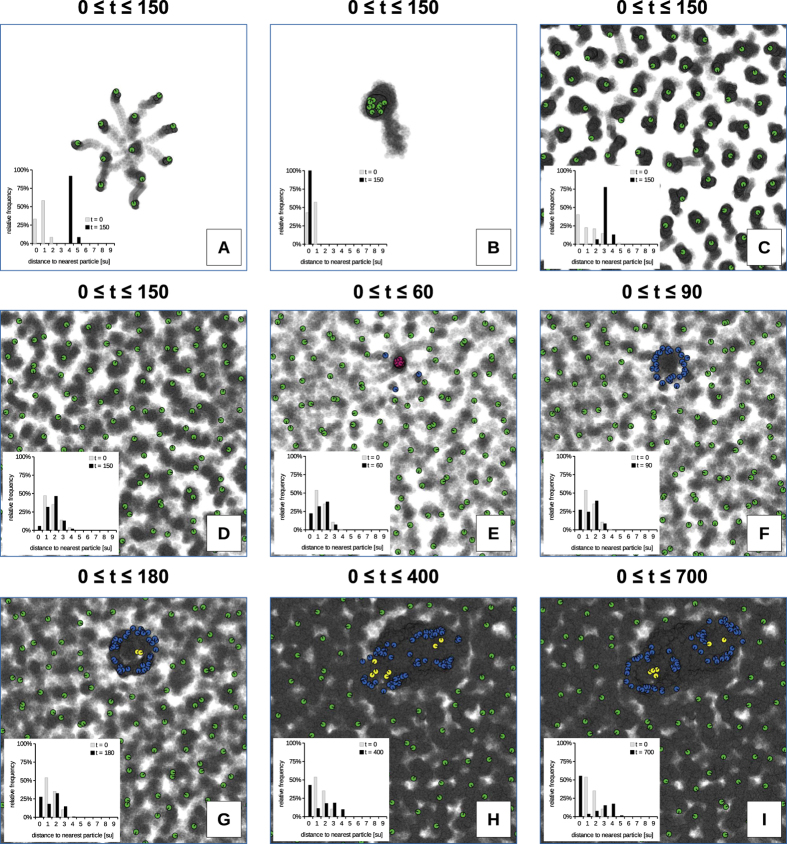
Collective behaviors of different particle numbers and densities. (**A**) 12 particles initially arranged around the habitat center. (**B**) 14 particles initially arranged around the habitat center. (**C**) Habitat filled with a density of *DPE* = *0.04 p*/*su*. (**D**) Habitat filled with a density of *DPE* = *0.07 p*/*su*. Subfigures (**A–D**) ran for 150 time steps. (**E–I**) Habitat filled with a density of *DPE* = *0.09 p*/*su* at time steps *t*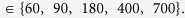 Semi-transparent grey background stamping shows older positions of particles, thus indicating trajectories of particles and (by their darkness) also past occupancy of areas. Other colors show local density of particles at the final time step, according to the color scheme indicated in the Method section and in Fig. 3. Each subgraph holds also a histogram of initial nearest-neighbor distances of particles and the final nearest-neighbor distance distribution at the end of the run in distance classes 1 space unit wide (1^st^ class: 0.00–0.99, 2^nd^ class: 1.00–1.99, …, 10^th^ class: 9.00–9.99).

For Fig. 2 we started first (Fig. 2A,B) a small group (*N*_*particles*_ ∈ {*12, 14*} particles) of particles randomly spread within 2.5 space units around the center of the habitat and ran it for 150 time steps. The first 149 positions of the particles were stamped semi-transparently in grey color to the ground to show the particles trajectories. The darker the grey color is there, the more often a particle was located there, and thus we also generated a “map of occupancy of space”. For the other subfigures (Fig. 2C,D) we kept this principle but filled the whole habitat space with certain Densities of Particles in the surrounding Environment (*DPE* ∈ {*0.04, 0.07*} *p*/s*u*) in a uniform random way concerning position and heading of particles. For Fig. 2E–I, we made snapshots at various time steps (*t* ∈ {*60, 90, 180, 400, 700*}) after starting with a randomized distribution of particles with a *DPE* = *0.09 p*/*su*. Each subgraph also holds a histogram of initial nearest-neighbor distances of particles and the final nearest-neighbor distance distribution at the end of the run in distance classes which are 1 space unit wide (1^st^ class: 0.00–0.99, 2^nd^ class: 1.00–1.99, …, 10^th^ class: 9.00–9.99).

**Figure 3:**
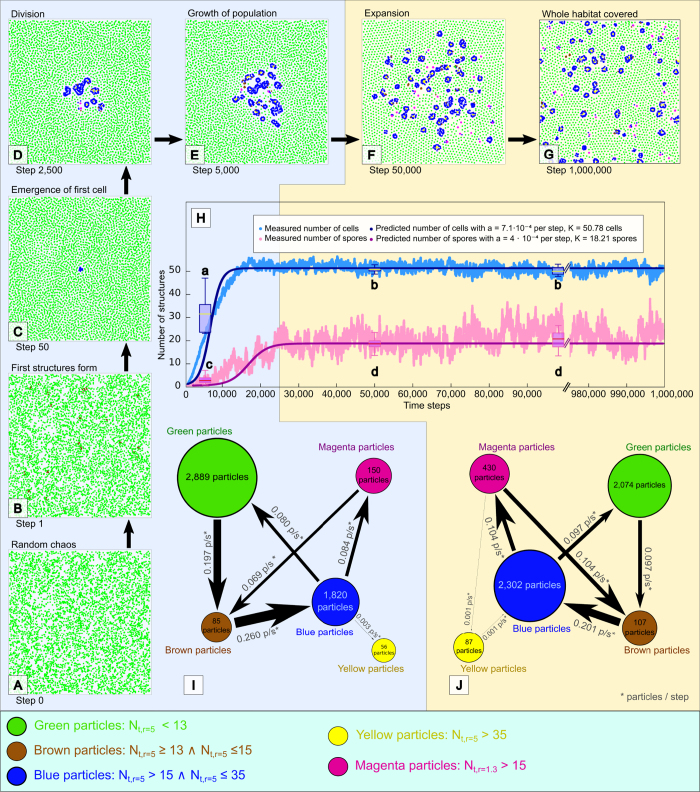
Spontaneous emergence and expansion of life-like structures into a density-regulated ecosystem. (**A–G**) Ordered structures appear, persist, replicate and spread from a randomly distributed and randomly oriented set of 5000 particles in 250 × 250 space units (0.08 p/su), observed for 10^6^ time steps. The emerging structures change the local density of particles, which is also expressed by particle colors. (**H**) Population dynamics of cells and spores follow a logistic (sigmoidal) growth pattern to which we modeled (minimum squared residuals) a classical macroscopic model of biological density-dependent growth^19^. Populations of cells and spores were analyzed from the number of blue/yellow and pink particles, based on the mean sizes of those structures (Supplementary Figure S3). (**I–J**) Observed rates of change of particles in the different local density states (color coded) during the growth phase of the sigmoidal growth (blue figure background) and during the saturated phase (yellow figure background). This represents a microscopic model of the emerging structures internal “physiological network”.

For the analysis shown in Fig. 3, we conducted 16 independent runs throughout 100,000 time steps (shown in Supplementary Figures S1 and S2) in a flat torus plane with 250 × 250 space units, containing 5000 particles (DPE = 0.08 p/su). One of these runs was selected randomly and continued until 1,000,000 time steps to investigate the long-term stability of population dynamics. These data were used for Fig. 3H–J.

We modeled a classical Verhulst-like-model^19^ of a density-dependent growth (*ΔX*/*Δt* = *a*·(*1* − *X*/*K*)·*X*) to the population of cells and spores (*X* models the population size) observed in this long run, as well as for all 15 short runs in which populations of emergent structures survived to the end of the run. We fitted the models by adapting the parameters *a* (maximum reproduction rate) and *K* (carrying capacity of the habitat) by applying the method of minimum squared residuals and applying an evolutionary algorithm to find minimum configurations (Fig. 3H). To estimate the number of cells and spores in all simulation runs, we assumed a typical size of a cell with 48 particles/cell and a typical size of a spore with 18 particles/spore, based on the analysis of those structures shown in Supplementary Figure S4. For the analysis in Figs 3I,J we recorded all transitions of particles from each color to all other colors, as well as numbers of particles in each color. Based on those recordings we identified the average cohort size of those particles as well as their average rates of change between colors during the “growth phase” of the population (*0* ≤ *t* < *25,000* steps; shown in Fig. 3I) and the “saturation phase” of the population dynamics (t ≥ 25,000 steps; shown in Fig. 3J).

**Figure 4:**
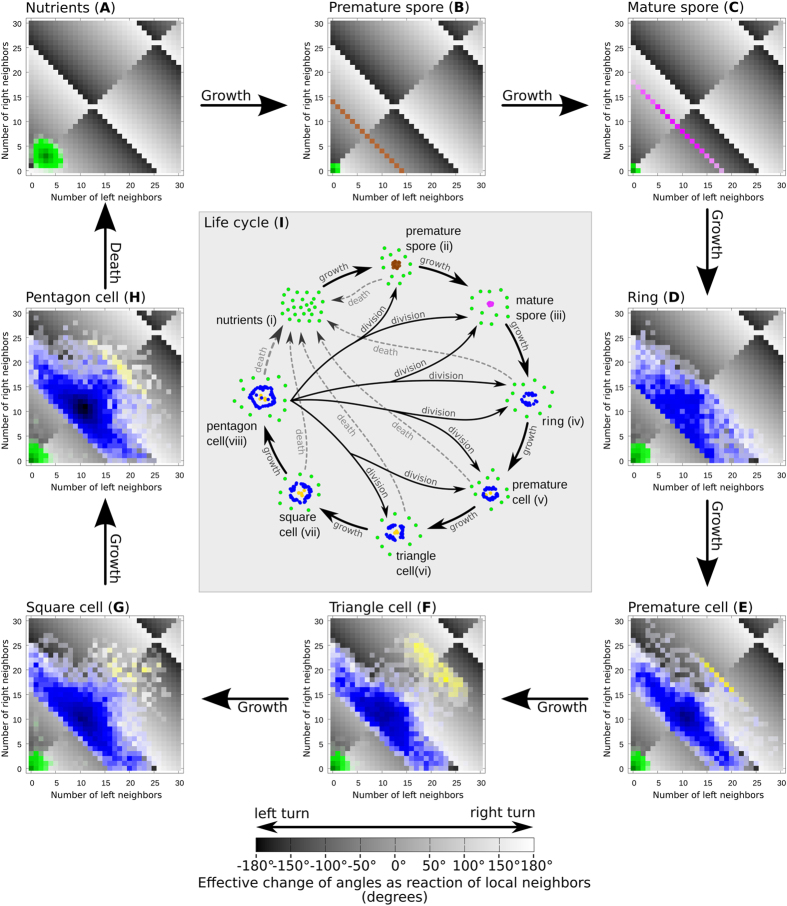
Heat maps in phase diagrams showing the time spent by particle in configurations with specific left-to-right (*L*_*t,r*_:*R*_*t,r*_) local neighborhood sizes within radius *r* = *5* (subfigures **A–H**). This determines particles’ rotation behavior (bright background: right turn, dark background: left turn) in our PPS. We observed particles building typical shapes in our PPS for 100 steps and display their overall turning regimes as a heat map so that the darker colors indicate higher frequency of these neighboring conditions, thus also higher frequency of this specific turning behavior. Characteristic structures reside for mostly in very specific neighborhood conditions. This suggests that the observed structures are self-stabilizing in a homeostatic way over long periods of time. Central subfigure (**I**): Resulting typical life-cycle of structures in *PPS* = 〈*r* = *5*, *α* = *180*°, *β* = *17*°, *v* = *0.67*〉.

For the analysis in Fig. 4 we imported typical structures that emerge in PPS (spores, cells of various size/age were “captured” previously in free runs of the model and were then “injected” into our analysis worlds) into an otherwise empty world. We observed for 100 time steps the left (*L*_*t,r*_) and right (*R*_*t,r*_) neighborhood size within radius *r* = *5* of every particle. For every particle in every time step this neighborhood configuration was looked up in a phase diagram of *L*_*t,r*_*:R*_*t,r*_ and a counter variable for this specific configuration was incremented by one. Finally all counter values for all neighborhood configurations were printed as a “heat map” over the phase diagram: The more often each specific configuration was observed, the darker the shade of the particle color was printed there. Every combination of left to right neighbors determines a specific turning angle according to equation 1, therefore the resulting turning angle for each neighborhood configuration was indicated as a shade of gray in the background of the phase plot, where dark colors indicate left turns and bright colors indicate right turns. Naturally, angles flip at 180° and 360° from left to right and vice versa. This property leads to the characteristic complex pattern of the figure backgrounds in Fig. 4A–H.

**Figure 5:**
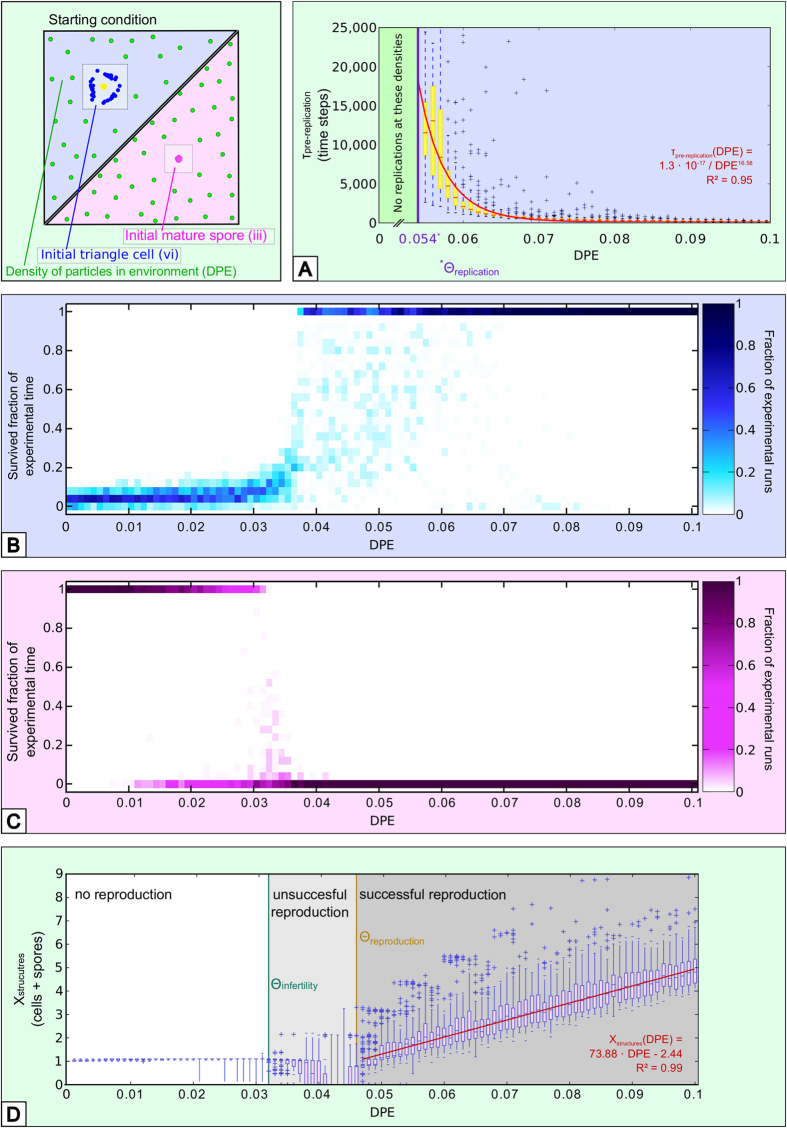
Microscopic parameters in dependence of environmental particle densities (DPE). (**A**) Time until replication of an initial triangle cell follows the regression function *τ*_*pre-replication*_(*DPE) = 1.3* · *10*^−*17*^/*DPE*^*16.58*^(*fit to the observed medians of the pre-replication periods per analyzed DPE setting*). (**B**) Initial triangle cells start to survive throughout 25,000 steps in a region of (*0.03* ≤ *DPE*), at lower environmental densities they lose particles and die. (**C**) Initial spore configurations survive for this time in environmental particle densities of (*DPE* ≤ *0.032*), at higher densities they grow out into cells. (**D**) Starting with a mature spore populations do not grow at densities below *DPE* = *0.032*, with *0.032* ≤ *DPE* ≤ *0.046* initial cells mostly unsuccessfully reproduce and die, while with DPE ≥ 0.047 final populations (*X)* start to grow linear with increasing DPE: *X*_*structures*_(*DPE*) = *73.88* · *DPE-2.44* for *DPE* ≥ *0.047* (*fit to the observed medians of the number of structures per analyzed DPE setting*).

For the analyses shown in Fig. 5 we used a 50 × 50 torus world with one of two characteristic structures (“triangle cell” and “mature spore”) initially placed within. The triangle cell used here consisted of 40 particles, the mature spore of 20 particles. All experiments were performed for 25,000 steps with 100 repetitions and varying densities of particles in the surrounding environment (DPE). We measured the time until cells replicate (division into two new cells, which eventually can grow into two copies of the primary cell, Fig. 5A). Spores always grow into cells, hence cannot replicate directly into two spore-copies and thus are not displayed in Fig. 5. We determined a successful replication by estimating the number of cells via particle counting. To make sure, that a replication took place only a cell-number estimation higher than 2.1 cells (instead of 2 cells) was considered a replication. We then fitted a graph to the median pre-replication periods applying the minimum residuals method using least squares. We also measured the number of steps until an initial structure vanished at varying densities of particles in the surrounding environment (which happens either through cell division or decay). The survived experimental time of a structure was discretized into 30 groups (Fig. 5B: initial triangle cell, Fig. 5C: initial mature spore). Darker shades of color represent more events in each of these groups. Finally we measured the number of structures (cells and spores) emerging from a single initially placed mature spore after 25,000 time steps (Fig. 5D).

**Figure 6:**
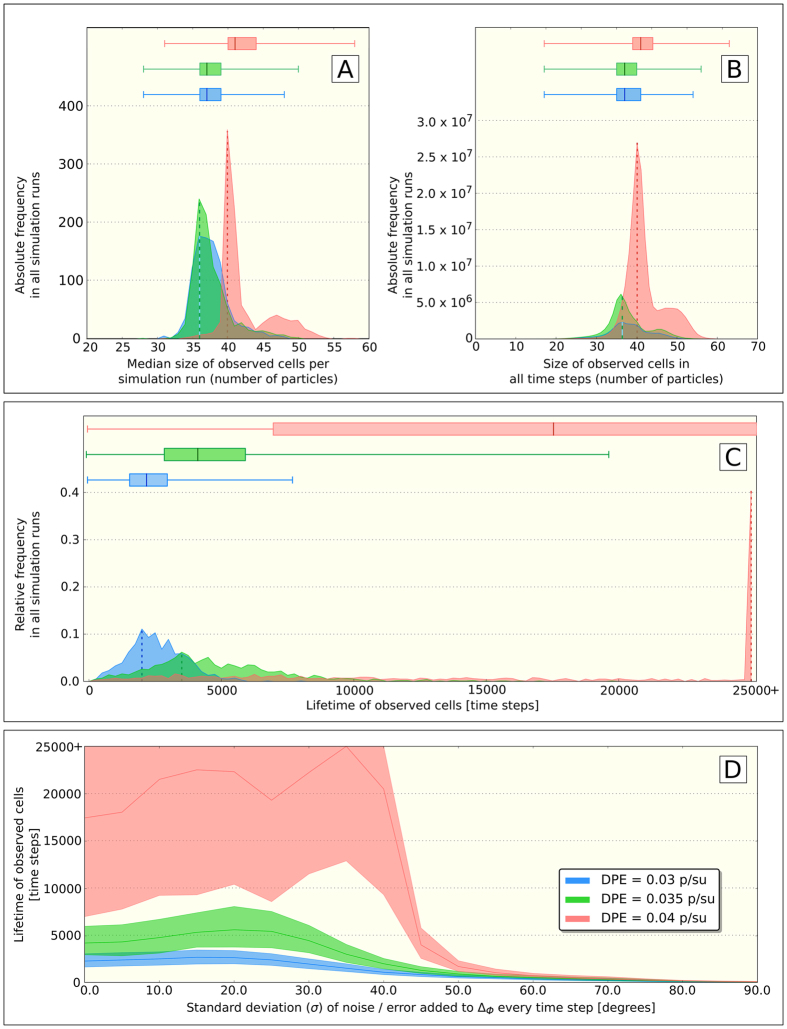
Further analysis of cell properties in dependence of environmental nutrient particle density (DPE = density of green particles in the environment) and in dependence of actuation noise/error. (**A**) Histogram of median effects of environmental particle density on the resulting size-distribution of cells within individual simulation runs (which were stopped when cells died). (**B**) Histogram of absolute effects of environmental particle density on the resulting size-distribution of cells across all performed simulation runs. (**C**) Histogram of effects of environmental particle density on the absolute survival (maximum lifetime) of cells across all simulation runs that lasted for 25,000 time steps. Colored areas show histograms of observed distribution, vertical dashed lines the most common observation (modus). Horizontal box-blots indicate minimum, maximum, quartiles and medians of the observed distributions. (**D**) Median (solid line) and IQR (filled areas) of effect of environmental density and actuation error (noise) on the observed lifetime of cells.

For the analyses shown in Fig. 6 a torus plane with 50 × 50 space units and surrounding particles (three different particle density levels: *DPE* ∈ {*0.03, 0.035, 0.04*} p/su) randomly placed on this plane were used for 1000 simulation runs. At the beginning of each simulation run we injected the same triangle cell (consisting of initially 40 particles) to the habitat, as it was already used in the analyses shown in Fig. 5. All simulation runs were performed for 25,000 time steps, measuring the cell size and the survived experimental time of each cell. In addition to our analyses of cell size distribution (Fig. 6A,B) and survivorship distribution (Fig. 6C), we investigated the impact of noise/errors to cell life expectancy (Fig. 6D). Therefore a normally distributed noise (η_*σ,t*_) was added to the change of turning angles of each particle in each time step, leading to the expression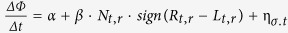, where we varied the standard deviation (σ) of the noise η_*t*_ in every time step *t*. This stability analysis was conducted with 1000 repetitions for each level of noise (σ ∈ {*0*°*, 5*°, …, *90*°}).

**Figure 7:**
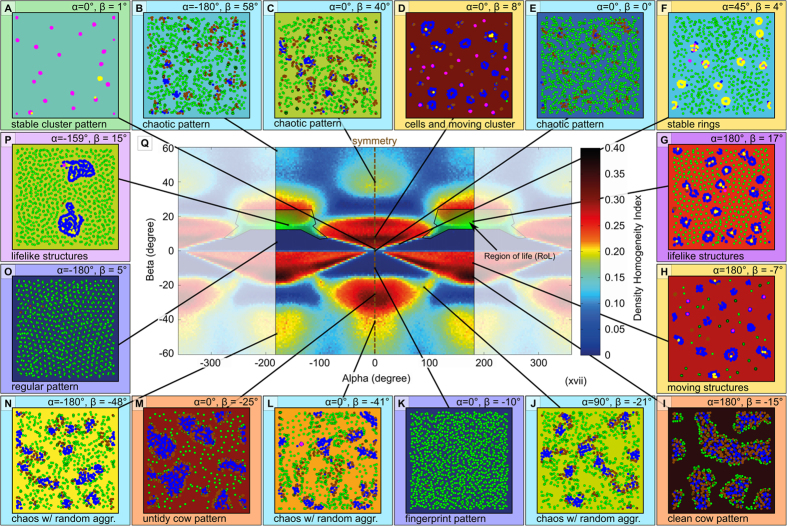
Parameter sweeps of possible PPS configurations in <α, β> parameter space. Systematic sweeps of *α* and β of PPS with v = 0.67, r = 5, density = 0.12 p/su. The central figure shows the resulting density-homogeneity-index after t = 500 steps (DHI (500)), where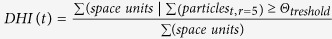. This scan shows a very distinctive set of regions in which particles are locally accumulated (yellow, red and black region), but only one specific region (“region of life”) was detected to produce self-replicating life-like structures.

For the analysis in Fig. 7 we used a 100 × 100 torus world with a particle density of 0.12 p/su. We performed a parameter-sweep of *−180*° ≤ *α* ≤ *180*° with an increment of *Δα* = *3.0*° and *−60*° ≤ *β* ≤ *60*° with an increment of *Δβ* = *1.0*°, repeating it 10 times per combination.

We introduced the density-homogeneity-index (DHI; shown in Supplementary Figure S5), contrasting the emergence of a wide range of potential interesting patterns within the PPS. The *DHI*(*t*) is characterized by the ratio of space units exceeding a certain threshold of particles (*Θ*_*threshold*_ = *14* particles) in their neighborhood (radius *r* = *5*) to all space units at time step *t*. Parameter combinations resulting in a low *DHI*(*t*) form uniformly distributed patterns, while high *DHI*(*t*) values indicate local accumulations of particles, which were then manually screened by us for visually identify emergence of life-like structures or other interesting patterns.

Runs with *α* = *0*° (symmetry-line) generate mirrored *DHI*(*t*) values and wrap at *α* = *180*° and *α* = −*180*°, thus mirrored and wrapped areas of the parameter space were marked by a pale overlay. Runs with *β* = *360*° show wrapped *DHI*(*t*), as they do also at *β* = *−360*° (not shown), however they have no symmetry at *β* = *0*°.

## Results

Figure 2 demonstrates the dependency of particle behaviors on their spatial density. A small group of 12 particles, which are started close to each other (see distance histogram), exhibit repellent forces and spread until they are separated from each other. However, they stay within interaction radius (*r*), thus are still actively sticking together as a group (Fig. 2A). In contrast to that, a group of 14 particles exhibits attractive forces that pull the group stronger together than they were initially placed. Then these forces move the whole group across the habitat as a hurricane-like premature spore (Fig. 2B). Filling the whole habitat with a low particle density (*DPE* = *0.04* *p*/*su*) makes all particles arrange in a hexagonal neighboring topology keeping contact with a median distance around 3.5 space units (Fig. 2C), while at higher densities (*DPE* = *0.07* *p*/*su*) the particles cannot arrange in a stable way anymore. In consequence they stay in motion in a way that is best characterized as “deterministic chaos” (Fig. 2D), exhibiting a wider spread of inter-particle distances (see distance histogram). Further increasing the density of particles (*DPE* = *0.09* *p*/*su*) produces again a “deterministic random” motion that eventually allows the local density to be high enough to form a premature spore at a random place. This premature spore moves locally and this way quickly attracts more particles to form a “mature spore” (Fig. 2E). This spore then quickly grows to a small ring cell (Fig. 2F), which further grows (Fig. 2G), moves slowly, and finally divides into 2 cells (Fig. 2H). Those two cells repel each other and further move slowly apart from each other (Fig. 2I). As the development of the inter-particle distances from Figs 1I–2E indicates the motion law of particles drives the system towards a specific near-equilibrium condition: A fraction of the particles are close together within cells, while the remaining free particles spread to a similar hexagonal-arrangement distribution as is found in Fig. 2A and C.

PPS initialized with randomized population (0.08 p/su, Fig. 3A) start to structure the habitat within a few time steps (Fig. 3B,C): First, a formation of a spiraling structure called ‘premature spore’ (Fig. 4B) emerges, which consumes free particles (‘nutrients’, Fig. 4A) to grow into a ‘mature spore’ (Fig. 4C), which then can further grow through a distinct life-cycle (Fig. 4I) into a ‘cell’. A cell can further grow and self-replicate until cells cover the habitat (Fig. 3A–G). This population grows sigmoidally (Fig. 3H) up to the point where nutrient particle consumption (growth) and release of particles through cell death (decay) are at equilibrium, as an ecological material cycle emerges in the system: Particles get recycled and often transformed in the internal physiology of the cell structures (Fig. 3I,J). The emerging macroscopic system shows self-ordering, homeostasis, self-replication, substance recycling, life-cycles and a self-creating ecosystem (Supplementary Video 1). Fitting a logistic-growth model to our observed population dynamics yields a reproduction rates of *a*_*spores*_ = *4.0* · *10*^−*4*^ per time step and *a*_*cells*_ = *7.1* · *10*^−*4*^ *per time step* and carrying capacities of *K*_*spores*_ = *18.21* spores and *K*_*cells*_ = *50.78* cells. Except the very beginning, populations of spores were found to be always smaller than populations of cells (Mann-Whitney U-test, N_1_ = N_2_ = 15, p < 10^−5^, Fig. 3H) and early populations were found to be significantly smaller than later ones (Mann-Whitney U-test, N_1_ = N_2_ = 15, p < 10^−5^, Fig. 3H).

A typical cell contains approx. 48 particles, a typical spore 18 particles. At the end of 15 experimental runs (*t* = *100,000 steps*) we counted on average 50.54 ± 0.36 (mean ± std.dev.) cells and 19.14 ± 1.32 spores prevalent. The Supplementary Figure S4 shows that spores start at 14 particles and contain up to 22 particles, yielding a mean number of 18 particles, while cells start at 23 particles and contain up to 60 particles, yielding a mean of 41.5 particles. However, we realized that cells grow fast and stay persistent for long, thus they exist longer in a grown-up state, and thus we used a particle-to-cell estimate of 48 particles/cell for our macroscopic cell population model depicted in Fig. 3H and Supplementary Figure S3.

Particles building characteristic structures cover specific regions in the phase-diagram of left (*L*_*t,r*_) versus right (*R*_*t,r*_) neighbors (Fig. 4A–H), thus will exhibit specific turning angles *Δϕ* according to equation (1). The resulting turning angle *Δϕ* is color-coded in the background of Fig. 4A–H as grey color shades for all combinations of *L*_*t,r*_ to *R*_*t,r*_ in the phase diagrams: Green ‘nutrient’ particles cover the left corner in phase-diagrams (Fig. 4A), performing mostly turns around ±180°. ‘Premature spores’ (brown) cover a sharp line (*L*_*t,r*_/*R*_*t,r*_) = [(*14*/*0*), (*0*/*14*)], see Fig. 4B. Particles (blue) of small ring cells cover mostly the triangular area between (*L*_*t,r*_/*R*_*t,r*_) = [(*16*/*0*), (*0*/*16*), (*14*/*14*)], see Fig. 4D. The yellow particles in “premature cells” form a sharp line (*L*_*t,r*_/*R*_*t,r*_) = [(*13*/*23*), (*23*/*13*)], see Fig. 4E. The most complex regime of particle configurations was found in “triangle cells”: The blue outer hull particles reside in a trapezoid region (*L*_*t,r*_/*R*_*t,r*_) = [(*15*/*3*), (*15*/*13*), (*13*/*15*),(*3*/*15*)], while the inner yellow particles reside within (*L*_*t,r*_/*R*_*t,r*_) = [(*15*/*23*), (*16*/*26*), (*25*/*15*), (*26*/*18*)], see Fig. 4F. Larger cell types show a preference to reside closely around (*L*_*t,r*_/*R*_*t,r*_) = (*11*/*11*). Spores (pink) exist in various types: Small spores reside on a sharp line (*L*_*t,r*_/*R*_*t,r*_) = [(*18*/*0*), (*0*/*18*)] (Fig. 4C). Medium spores, (*L*_*t,r*_/*R*_*t,r*_) = [(*38*/*0*), (*0*/*38*)], and large spores, (*L*_*t,r*_/*R*_*t,r*_)* = *[(*58*/*0*), (*0*/*58*)], show similar shapes (data not shown). We assume that there exist an endless number of such mega-spores and also other regimes for yellow particles in very high densities of particles, which allow no self-replicating cells to emerge anymore.

Besides investigating the microscopic processes of individual particles (Figs 3I–J, 4), we also studied characteristic parameters of initially seeded structures (cell and spore) at various densities of particles in the surrounding environment (DPE). We found that there is a critical density of particles (*Θ*_*replication*_ = *0.054 *p/su, Fig. 5A) below which cells don’t replicate. The higher DPE gets above this threshold, the faster reproduction is (Fig. 5A). Cells in a DPE below 0.03 p/su rarely survive for 2500 steps (Fig. 5B), while spores above 0.032 p/su grow to cells (Fig. 5C). We found a critical threshold (*Θ*_*infertility*_ = *0.032* *p*/*su*) below which initially seeded structures don’t try to reproduce (Fig. 5D). At DPE above *Θ*_*reproduction*_ = *0.046* p/su cells replicate successfully to populations that scale linearly with DPE (Fig. 5D). In DPE between *Θ*_*infertility*_ and *Θ*_*reproduction*_ cells often die due to unsuccessful replication tries.

For further characterizing our cell populations we analyzed the size distribution and the survivorship-distribution of cells by starting a typical triangle cell of 40 particles into randomized habitats of varying environmental particle density (*0.03* ≤ *DPE* ≤ *0.04 p*/*su*): Low environmental nutrient densities (*DPE* = *0.03* *p*/*su*) allow cells to survive mainly between 1,720 and 3,116 time steps as is characterized by the inter-quartile range (IQR), showing a median lifetime of 2,346 time steps and most cells living for 1,888 (modus) time steps (Fig. 6C). With medium nutrient density (*DPE* = *0.035* *p*/*su*) cells survive between 3,006 and 6,016 time steps (IQR) with a median lifetime of 4,248 time steps and most cells surviving for 3,470 (modus) time steps (Fig. 6C). With high nutrient density (*DPE* = *0.04* *p*/*su*) cells survive between 7,037 and 25,000 time steps (IQR) with a median lifetime of 17,450 time steps and most cells surviving for 25,000 (modus) time steps (Fig. 6C).

With *DPE* = *0.03 p*/*su* and *DPE* = *0.035* *p*/*su*, we found that the size distribution of cells to span between 36 particles and 39 particles (Fig. 6A) when looking at median cell size per run, and between 35 particles to 41 particles per cell (Fig. 6B) when considering all cell sizes in all time steps in all simulation runs. This difference is due to the fact that with medium environmental particle density more cells are observed for more time steps than with low free particle density (Fig. 6B). In both environmental configurations the median size in both analyses is 37 particles per cell (Fig. 6A,B) and the most common cell size (modus) is 36 particles. These size spans correspond to cell shapes ranging from small ring-shaped cells to small triangle-shaped cells (Supplementary Figure S4), the median observed cell size represents a large ring-shape cell (Supplementary Figure S4). With high environmental particle density (*DPE* = *0.04* *p*/*su*) we observe cell sizes to span between 40 and 44 particles (IQR, Fig. 6A), when looking at median cell sizes per run and 39 particles to 44 particles (IQR, Fig. 6B) when looking across all time steps in all runs. This size span corresponds to triangle-shaped cells of various sizes (Supplementary Figure S4). The median cell size at this condition is 41 particles and the most common cell size (modus) is 40 particles (Fig. 6A,B) for both analyses (medians of runs and across all time steps in all runs) what represents a large triangle (compare Supplementary Figure S4).

To analyze the stability of the emergent cell structures in our PPS we analyzed the survival period of an initially placed cell of 40 particles in initially randomized habitats of varying environmental particle density (0*.03* *≤* *DPE* *≤* *0.04* *p*/*su*) by adding normal-distributed rotation noise to the second term of the right-hand side of equation 1. The noise was symmetrically distributed around 0°, with varying standard deviation (*σ*) and ceiled/floored between −180° and +180° for the extremes. Figure 6D shows that the density dependent-life times already observed in Fig. 6C for the 3 tested environmental situations (DPE levels) are slightly enhanced by noise up to *σ* = *30*° (low and medium DPE setting) and *σ* = *40*° (high DPE setting) and that cells can exist only for a few hundred time steps with higher noise levels.

To assess how ‘special’ the *PPS* = 〈*r* = *5*, *α* = *180*°, *β* = *17*°, *v* = *0.67*〉 is, we analyzed the whole family of *PPS* = 〈*r* = *5*, *α* = *, *β* = *, *v* = *0.67*〉 at *DPE* = *0.12*. Figure 7Q (center) shows that most resulting behaviors of those PPS are very distinctive from our focal PPS (and its close neighbors in 〈*α,β*〉 parameter space). To quantitatively analyze each PPS we calculated how many units of space showed a Density Homogeneity Index above a specific threshold *Θ*_*threshold*_ = 14 particles, thus 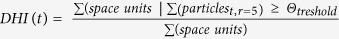 to give an index of spatial homogeneity. PPS that showed high values in this density-homogeneity-index were then screened manually to qualitatively describe their behaviors (Fig. 7A–P).

## Discussion

The simple motion law we present here (Fig. 1) enables the emergence of life-like structures (cells and spores) which self-reproduce and grow in a life-cycle (Figs 2, 3 and 4I). These structures build an emergent ecosystem (population) on the macroscopic system level purely based on the microscopic rule set described in equation 1. These populations of cells show dynamics that are well captured by the logistic growth model (Fig. 3H) known to describe also the growth of natural organism populations. We discovered another ecosystem property in the “nutrient cycle”: The emerging cells show a certain intake and loss of free “nutrient particles” while they sustain their presence in the habitat (Figs 2E–I and 3I,J). We show that growth, survival and reproduction depend on the environmental habitat situation (free particle density, Fig. 5), which also affects the size and the age distribution of cells, which is the ultimate consequence of density-dependent longevity of individual cells (Fig. 6A–C). Instability analysis showed that the observed life-like structures are not just very specific fragile mathematical artifacts (Fig. 6D). In contrast, they emerge and survive even in presence of high levels of noise in the individual conduction of the microscopic motion law.

It is unknown yet, why this specific PPS produces such an interesting and rich set of nature-like structures, thus we expect further analysis may yield fundamental understandings in mathematics and algorithmics, as it was also the case with specific cellular automata^16^ and fractals^20^. We identified a specific “region of life” (RoL) in the 〈*α*, *β*〉 parameter space containing such life-like structures (Fig. 7). However, it is neither understood why they appear especially there nor is it clear how the RoL changes with particle speed or interaction radius yet.

The primary aim of this article is to showcase this extremely interesting PPS in the configuration *PPS* = 〈*r* = *5*, *α* = *180*°*, β* = *17*°, *v* = *0.67*〉 to assess its key properties and highlight its internal mechanics. Although it is not fully understood yet why exactly this microscopic motion law produces such a rich and life-like macroscopic system we can already describe the main basic mechanisms and properties of the system here based on the observations and analyses we made in the study: In the PPS the most interesting structures, which are cells and spores, emerge by self-organization from a simple mechanistic microscopic motion law.

How do cells and spores emerge? How do they grow and reproduce? At locations of (randomly happening) higher density of particles (14 particles in each other’s interaction radius *r*, see Figs 2A,B and 4B and Supplementary Figure S4) a premature spore can form and further attract particles to grow into a spore. A spore is a compact assembly of particles (approx.16–21 particles in each other’s interaction radius *r*, see Figs 2E and 4C) that spiral around each other in close vicinity. Such an aggregate attracts additional free particles due to the motion law. At first, this is just a self-reinforcing process (positive feedback) that leads to the observed growth of the spore. At a certain size threshold of 23 particles (see Supplementary Figure S4) this positive feedback loop lets the spores “germinate” to cells (see Fig. 2F) as the newly attracted particles rotate more than 180° to the aggregation side (Fig. 4A–H), thus they start to turn away from the spore’s center. In fact, the positive feedback has turned into a negative feedback at this point of growth. After that, the next stable state is the “cell” state, which can consist again of a rather dense core (yellow particles), an almost empty inner space region and a dense outer “membrane” layer (blue particles), see Fig. 2G. Further intake of particles makes the cells grow to a certain size, simultaneously increasing also the intrinsic pressure of particles, as particles repel each other at specific neighborhood sizes. At a certain size of approximately 61 particles, depending on the spatial configuration and internal pressure, the cell can break into 2 cells (see Fig. 2H,I) or produce one or two new spores that are thrown out of the compound or divide directly into 2 new cells (see Supplementary Figure S4). For successful replication the environment has to have a high enough density of available free nutrient particles (see Fig. 5D).

Do cells change their habitat? Can they be seen as a thermodynamic equilibrium configuration? A cell in a PPS consists of areas of high and areas of low particle density, thus they have a characteristic internal density inhomogeneity. The phase plots in Figs 4B–H show that the observed structures (spores and cells) steadily increase the overall local density of particles inside of them as it is also demonstrated by the analysis based on DHI(t) in Fig. 7 and by the histograms shown in Figs 2E–I. This indicates that cells consume free particles from their surrounding during their initial growth and their later reproductive period which is most prominent in times when populations are on the rise (See Fig. 3I). The equilibrium size of cells depends on the density of environmental particles available to consume (Fig. 6A,B). This nutrient consumption ultimately decreases the free particle density in the environment, as thus particles represent a limited shared resource for cells. Over time, the free particle distances increase, as the pressure in the environment goes down, while pressure increases inside of the cells, as can be seen by the bimodal distribution in the histogram of Fig. 2I, Finally, the “life expectancy” of cells depends on the availability of free nutrient particles, what in turn limits the population growth and survival when those particles get scarce (Fig. 6A–C). However, cell death can replenish available free particles again which reshuffle in the environment over time and allow cells to prosper later at other regions of the habitat. This then closes the emergent nutrient cycle in the system.

In ecology, this would be considered to be a case of intra-specific competition for food. The fact that a species limits its own growth by depleting its environment of available food was found, as an exemplary case, in seabirds^21^. Such a self-limitation of available free particles finally creates another negative feedback in the system, which prevents a cell from further growth and reproduction. Thus, cells reside at the equilibrium of positive feedback (attraction), and negative feedback (scarcity of free particles and active expelling surplus particles due to high intrinsic pressure), as is shown in Fig. 3J. While spores represent a rather stable and simple steady state, cells represent a more complex and more dynamic steady state of the system. This system description is a high-level understanding of the functioning of cells we can deduct from the findings presented here. However, for a full understanding of the system, it requires to completely close the causal link between the microscopic parameters of the motion law and all observed macroscopic features of the system. Ultimately, as also Fig. 6 already partially demonstrates, all macroscopic properties of the system (size and age distribution of cells, shape of cells, ratio of cells to spores, population size/density and growth rate, speed of reproduction, etc.) depend on the 4 parameters *α*, *β*, *r* and *v*. Parameters-sweeps of *α* and *β* already indicate dramatic phase-transitions in those macroscopic properties with minimal changes of microscopic parameters (Fig. 7) while in other regions of parameter space there are large plateaus that keep macroscopic properties robust. A deep understanding and reasoning of this is calling for future studies, which are currently in the making.

How does the microscopic motion law rely to physical or chemical systems? On the first sight our motion law with *α* = *180*° looks counterintuitive, as it leads to particles that steadily oscillate in their orientation in a back-and-forth way. However, this is not so counterintuitive as it first looks: This oscillatory behavior makes a particle stop in places without any neighbors and makes it rotate around a local spot with low density neighborhood. All other possible configurations of any SPP that binds the particles’ turning angles purely to local neighborhood density will yield similar behaviors, just for different local densities of particles, for any give value of *α* (even for *α* = *0*°). A particle in a *PPS* = <*α* = *180*°, *β* = *17*°> behaves similar with a neighborhood size of *N*_*t*_ = *0* particles compared to a particle in a *PPS* = <*α* = *0*°, *β* = *17*°> with a neighborhood condition of *N*_*t*_ = *180*/*17* particles. Both particles will oscillate and thus stay in place. When we look at the emerging long-term behavior of a particle in our system, it shows that a particle stays in place when it is isolated, as it requires interaction (forces) with other local particles to be kept in motion. Thus, in the long-timescale view our system can be understood as a physical dissipative system. This makes our PPS comparable to e.g. a population of molecules that are dissolved in a fluid. Such molecules will also stop their motion without being kept in motion by interaction with nearby (e.g. water) molecules due to temperature (Brownian motion) or current flows. Thus, our PPS motion law uses a SPP system in a way that it is closer to the situation proposed for the origin of life than a more classical SPP system (that would have *α* = *0*°), which models rather the trajectory of a photon moving through friction-less vacuum space. SPP models with *α* = *0*° seem to be better suitable for modeling collective behaviors of agents (animals) that move actively by themselves and rather not for floating molecules in a primordial soup and hence call for a value of *α* = *180*°.

How does the PPS observed here relate to the conditions assumed at the origin of life? There are several significant resemblances between the PPS and the conditions that are thought to be valid for the origin of life on earth: We found that in the PPS spontaneous emergence of ordered self-replicating structures emerges only above a threshold density of components (pressure) and only above a certain speed threshold (temperature) starting from a randomized initial distribution. Similar properties were shown empirically to lead to the spontaneous emergence of the building blocks of life in chemical experiments mimicking earth’s condition at the time of the origin of life^22^. Such conditions are still found at hydrothermal deep-sea vents, which are discussed as promising candidates for the places of the origin of life^23^. Further aspects of the PPS (noise tolerance, continuous space, continuous asynchronous motion, local interaction) are furthermore very likely properties for the system in which real life emerged on earth.

How does this study relate to other works in the field? Self-structuring and pattern formation arising from random initial states are often modelled by reaction-diffusion models (RDM)^24^ or particle models, such as self-propelled particle systems^11^. Morphogenesis in such systems was explored in more complex SPP systems^25^,^26^: These particles can sense a continuous potential field, linking the microscopic SPP system back to the macroscopic RDM. Our PPS works without such fields, thus it is fully microscopic, still yielding high macroscopic complexity like self-replication of structures. There are several outstanding differences of our PPS to existing SPP models: Direction-alignment happens in an emergent way without requiring that particles sense the orientation of their neighboring particles, compare^11^,^27^. Particles do not require blind spots in perception, compare^28^, and particles need not to sense the center of mass of the local structure they are a part of, compare^29^. Our PPS model does also not require particles to sense the distance to their neighbors, not even in discrete distance zones, compare^27^,^28^,^30^. It is also not necessary to sense and compute any global field (e.g. potential fields) as they are often used in SPP, compare^25^,^31^,^32^. Still, PPS achieve comparable macroscopic system behaviors by forming emergent structures without requiring the sensor model usually found in classical published models of flocking, shoaling and swarming. Thus, PPS are simpler in their microscopic rules, yet still producing outstanding phenomena.

The common ancestor of life-forms is assumed not to be a “modern cell”^33^. This motivates minimal-cell models^34^ and protocell models in mathematics^35^ and chemistry^36^. Simple embodied non-genetic cell-like structures exhibit behavior, interaction in vicinity, and even duplication^37^. Thus, such pre-biotic compounds might be interpreted as SPP systems, showing even self-assembly after adding ssDNA for selective anchoring. In relation to other protocell models that mimic certain properties of living cells, especially droplet systems^37^ share many characteristics with our primordial particle system. Oil-in-water droplets or camphor disks on water surfaces show not only self-movement, they can also alternate between rest and motion phases^38^. Similar to that, primordial particles are able to remain in a certain position by oscillating back and forth, as well as they are able to move directionally in a place with adequate neighborhood composition. Nitrobenzene droplets in aqueous environment seeded with CTAB (cetyl trimethylammonium bromide) can divide themselves into smaller stable aggregates without external forces^39^. While primordial particles also exhibit this behavior, daughter cells even start absorbing nutrients, grow and finally divide themselves, leading to a potential infinite dispersal. Other oil droplets in water systems are known to generate spontaneous mode switching^40^, as do primordial particles in accordance to their neighborhood). While all of these models show stunning properties separately, a droplet system that combines all these characteristics is to be discovered yet like the primordial particles system does.

What open questions call for further investigations? In this article we describe the novel PPS that is capable to exhibit life-like properties. We analyzed several microscopic and macroscopic system properties. Still many fundamental questions are waiting for further investigations, which are beyond the scope of this first article: We think it will be extremely interesting to find the exact causal linkage between the macroscopic phenomena and the microscopic rules. The system will be only fully understood if somebody can explain for example why a system with *β* = *17*° (up to *β* = *25*°) produces reproducing cells while a similar system with a *β* = *26*° does not (Fig. 7) with our standard settings of *DPE*, *α*, *r* and *v*. For example, meta-models should predict how the five core parameters affect macroscopic properties like cell density or the equilibrium ratio of spores to cells. Obviously other values of *α*, *β*, *r* and *v* can yield other interesting systems (universes) that wait for their discovery and analysis (see Fig. 7). By allowing heterogeneous systems, which is mixtures of particles, more elaborate PPS can enter the field of artificial chemistries^41^ from a simple approach in which interactions are emergent instead of being pre-programmed. We predict such systems to produce even more fascinating collective systems.

What are the implications of these novel findings? We expect that the simplicity of the motion law and the system’s robustness against noise and error will allow physical manifestation, e.g. in active matter installations built by swarms of autonomous (likely nano-robotic) units. Such particles will also be quite limited in what they can sense about their local neighbors, they will not be able to actively communicate across distances, they will have limited computational power and they will be subject to sensory noise and actuation error. The fact that our PPS does not require much concerning individual capabilities of particles makes it an ideal candidate for novel “active matter” systems, especially on the microscopic scale. But also on the larger size scales the fields of swarm robotics^42^ and modular robotics^43^, self-reconfiguring robotics and self-healing robotics can profit from the power of this simple rule of interaction of the PPS. As self-reproduction is one of the key features of the PPS, also evolutionary swarm robotics are expected to profit from these novel findings.

In PPS, particles resemble configurations and dynamics of physical matter states: Start from gas-like random configuration, the system self-organizes into a rather regular-spaced ordered regime, similar to solid crystals. Cells and spores show a median connectivity of particles and flow dynamics, thus resemble a liquid state of matter: In PPS, life-like structures emerge from a phase-transition. However, when looking at green particles in time-lapse on long time-scale they exhibit fluidic dynamics, like mineral soils appear solid on short but fluidic on long scales.

Here we present a self-complexifying system as a model of protocells. Comparable systems governed by simple interaction laws exist on various spatiotemporal scales: Runaway galaxies and stars, equivalent to our occasionally appearing moving spores, and black holes, like our stable spores^44^. Growing galaxies and their resulting ratio of galaxy-bound to halo-forming suns are comparable to our PPS, as are also animal swarms^45^, and, hypothetically, also molecular, atomic and subatomic entities^46^. Here we focus only on structures emerging in one specific PPS. However, there are endless configurations for PPS, thus other “PPS universes” might yield many more fascinating creatures and structures awaiting their discovery.

## Additional Information

**How to cite this article**: Schmickl, T. *et al*. How a life-like system emerges from a simple particle motion law. *Sci. Rep.* **6**, 37969; doi: 10.1038/srep37969 (2016).

**Publisher’s note:** Springer Nature remains neutral with regard to jurisdictional claims in published maps and institutional affiliations.

## Supplementary Material

Supplementary Information

Supplementary Video
