# Corrigendum: How a life-like system emerges from a simple particle motion law

**DOI:** 10.1038/srep42454

**Published:** 2017-02-21

**Authors:** Thomas Schmickl, Martin Stefanec, Karl Crailsheim

Scientific Reports 6: Article number: 37969; 10.1038/srep37969 published online: 11 30 2016; updated: 02 21 2017.

In the original version of this Article, all instances of “simple” were incorrectly given as “simplistic”. This error has now been corrected in the PDF and HTML versions of the Article.

