# Supplementary material for: How a life-like system emerges from a simple particle motion law
Source: Sci Rep. 2016 Nov 30;6:37969. doi: 10.1038/srep37969 (PMC5346932; doi:10.1038/srep37969)
Supplement: Supplementary Information [file srep37969-s1.doc]

**Supplementary information**

**How a life-like system emerges from a simplistic particle motion law**

**Authors:** Thomas Schmickl^1^*, Martin Stefanec^1^, Karl Crailsheim^1^

**Affiliations:**

^1^ Department for Zoology, Karl-Franzens University Graz, Austria.
 *Correspondence to: [thomas.schmickl@uni-graz.at](mailto:thomas.schmickl@uni-graz.at), +43 316 380 8759

**Supplementary figures**


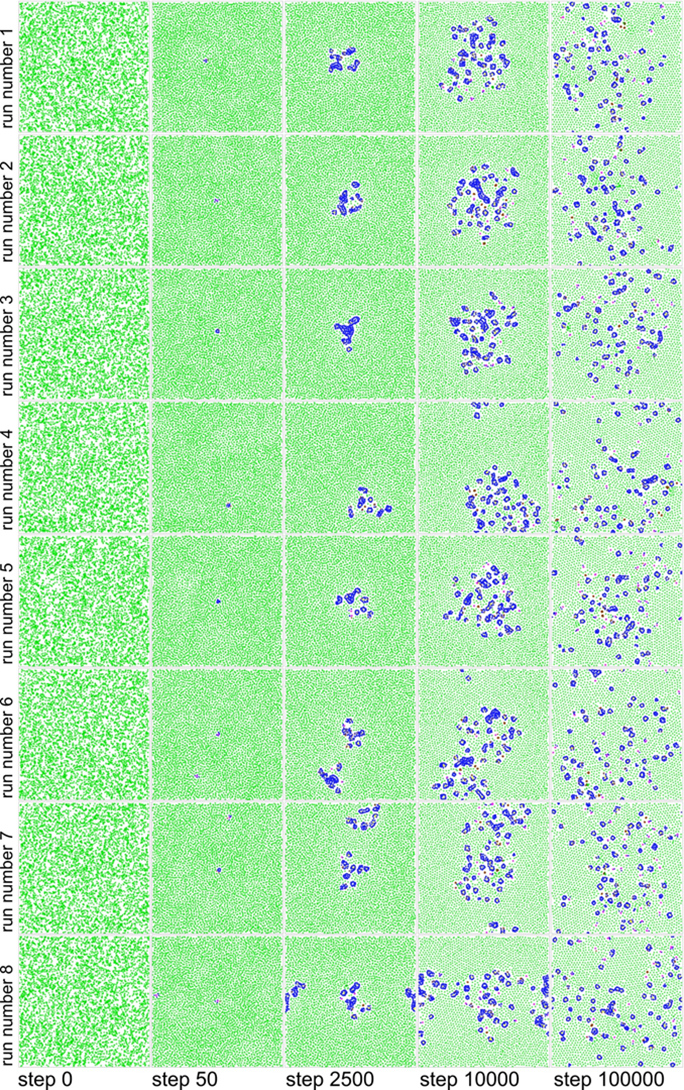


**Fig. S1.** **Emergence of life-like structures**: 8 independent runs with 5,000 particles randomly distributed and randomly oriented in a space of 250 by 250 space units. All runs lasted for 100,000 timesteps and in all runs patterns emerged at very early timesteps (*t<50*) followed by a period of growth and reproduction.


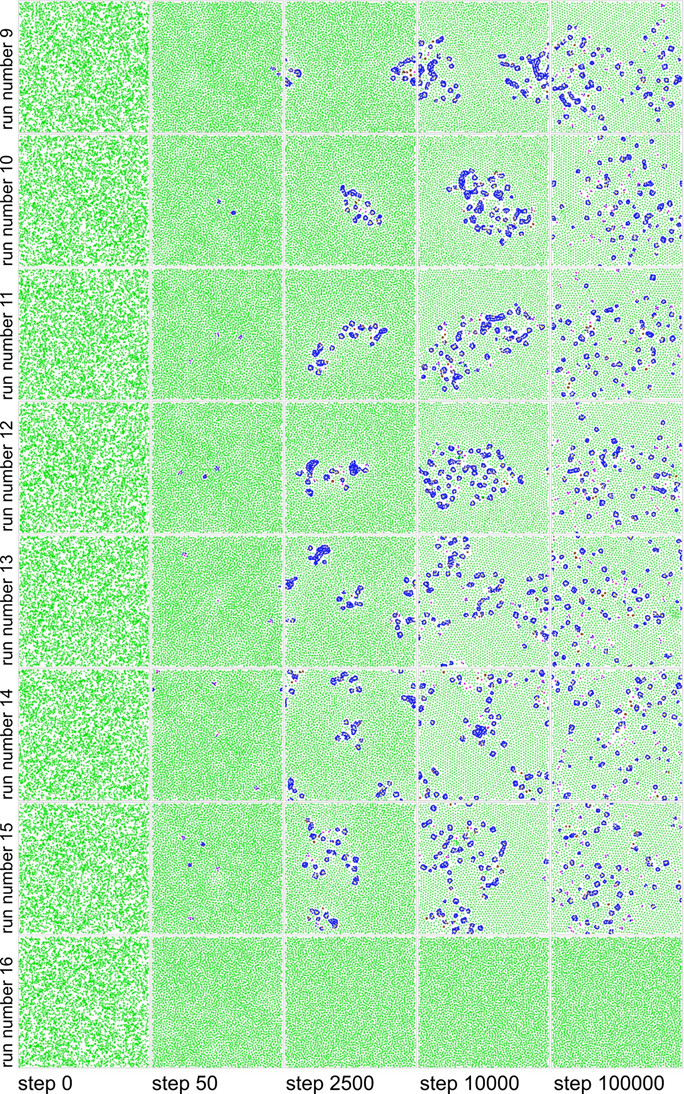


**Fig. S2.** **Emergence of life-like structures**: 8 independent runs with 5,000 particles randomly distributed and randomly oriented in a space of 250 by 250 space units. All runs lasted for 100,000 timesteps and in 7 of 8 runs patterns emerged at very early timesteps (*t<50*) followed by a period of growth and reproduction.


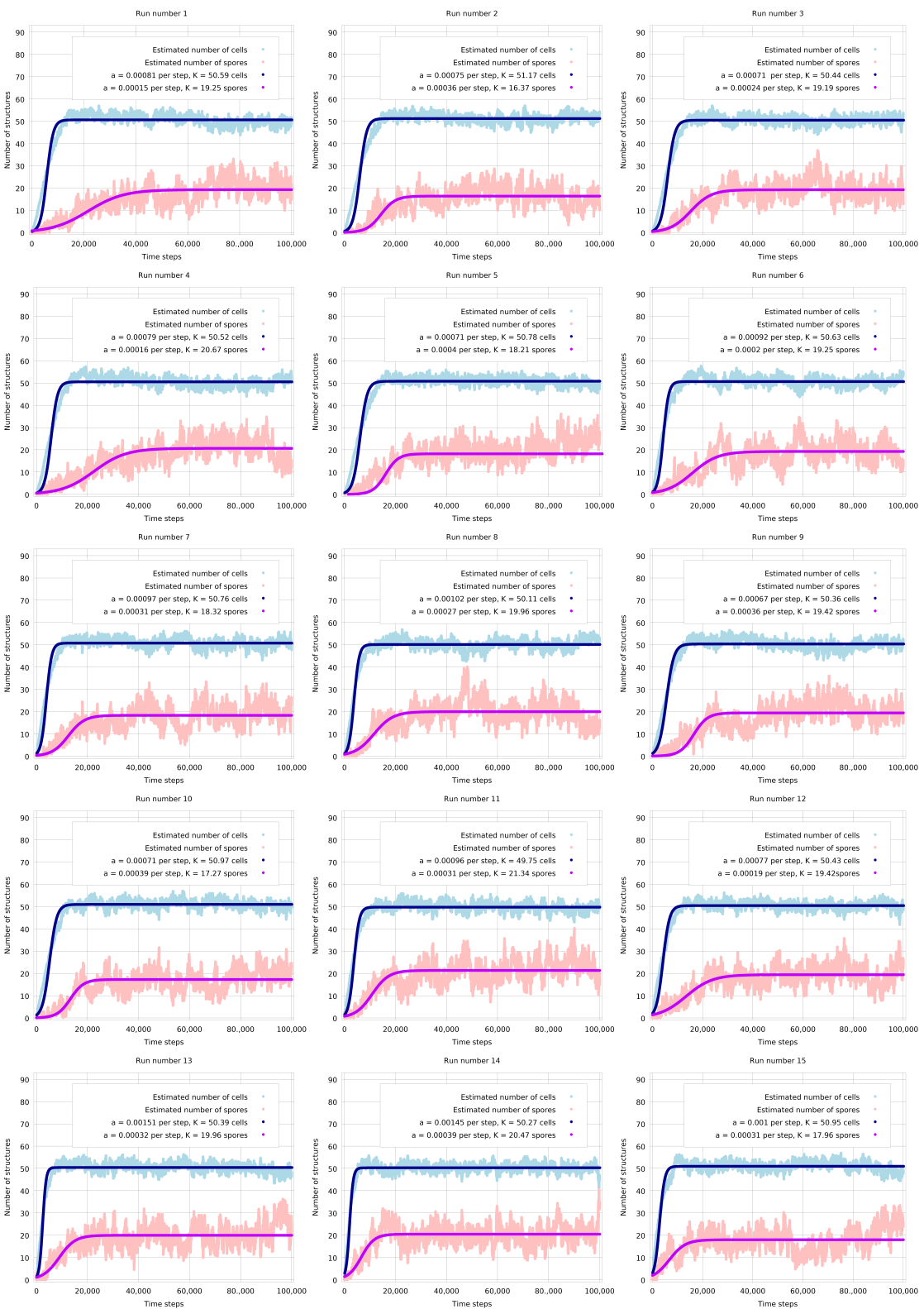


**Fig. S3.** **Comparing population dynamics of cells and spores in 15 repetitions** (cf. Fig. S1 and S2). Cells and spores follow a logistic (sigmoidal) growth pattern to which we modeled (minimum squared residuals) a classical macroscopic model of biological density-dependent growth: *ΔX/Δt=a⋅(1-X/K)⋅X*.


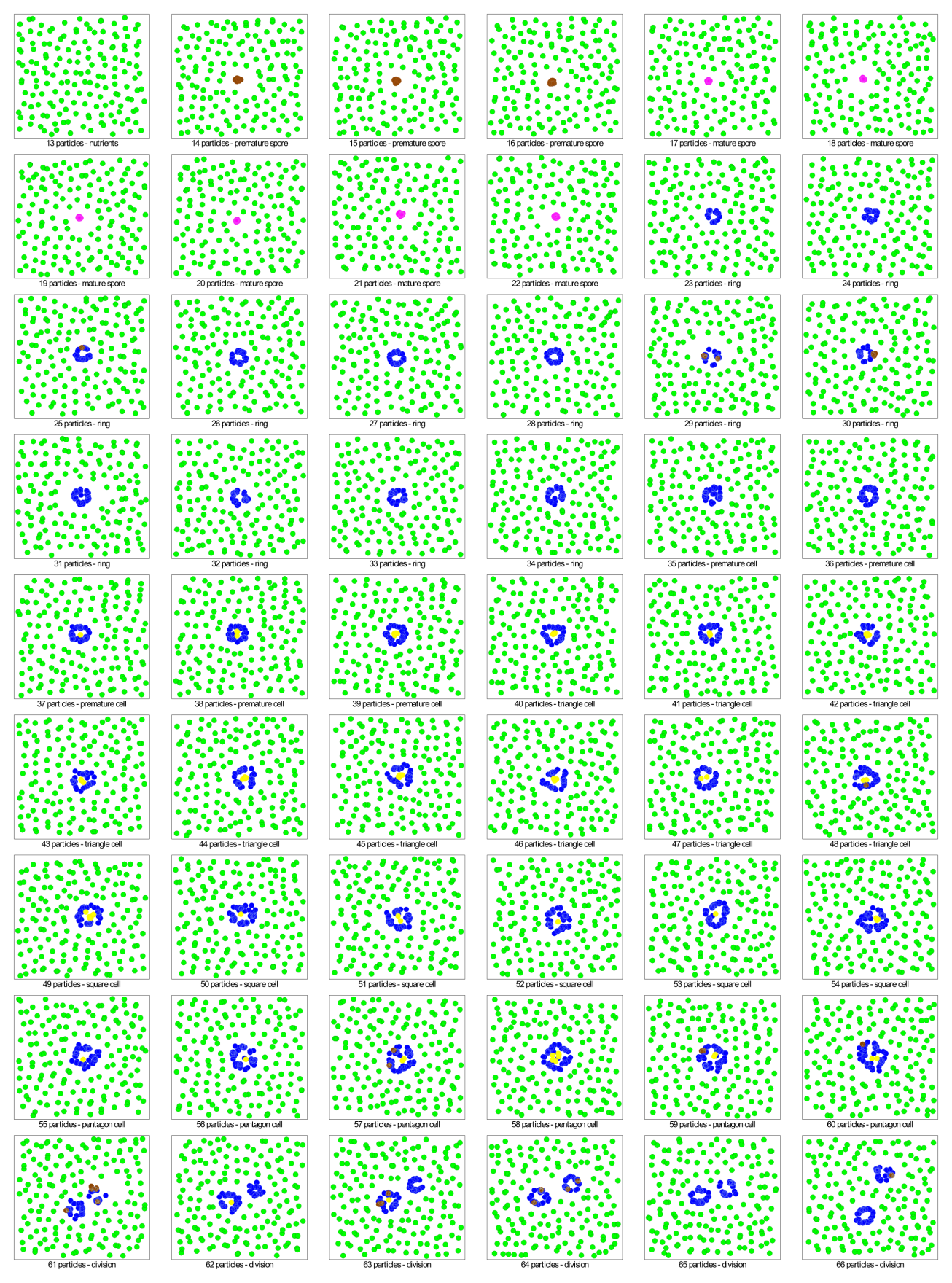


**Fig. S4.** **Typical structures occurring in *PPS=⟨r=5,α=180°,β=17°,v=0.67⟩*** . Structures and number of particles they contain.


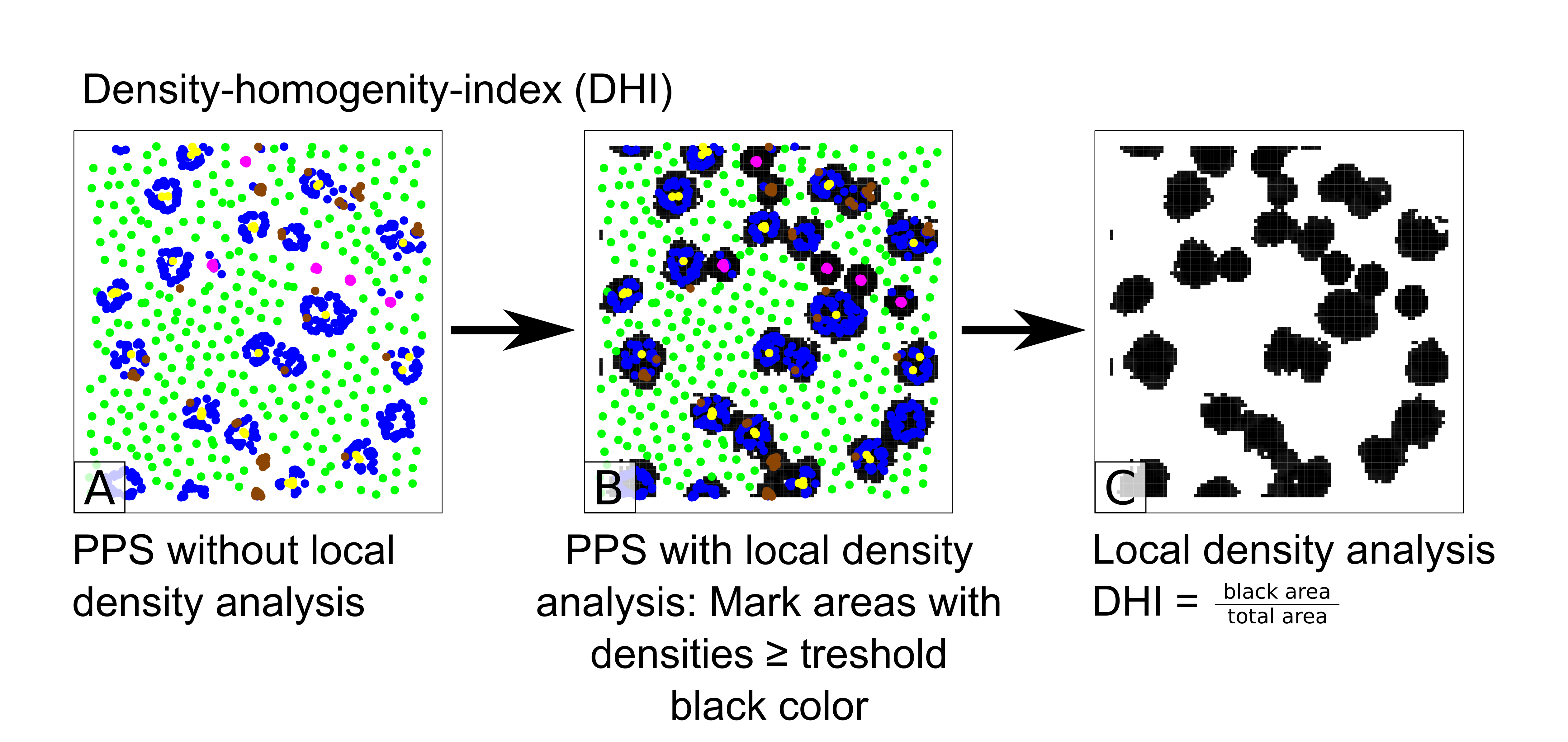


**Fig. S5.** **Density-homogeneity-index**: (A) a typical PPS pattern at *PPS=⟨r=5,α=180°,β=17°,v=0.67⟩* at a density of 0.12 p/su, (B) marking space units with more than 14 particles in radius 5, (C) ratio of marked space units to all space units equals the density-inhomogeneity-index

**Legend of the supplementary video file “How life emerges from a simplistic particle motion law.mov”:**

This video shows the basic rules of our motion law. It highlights the most important features of the emerging life-like structures and the ecology they create. It briefly shows the main results (graphs) of the paper and gives an outlook on other structures that can be found in the primordial particle system. Music source: Chris Zabriskie via freemusicarchive.com, 2012, released under an attribution license (<https://creativecommons.org/licenses/by/4.0/>), <http://freemusicarchive.org/music/Chris_Zabriskie/Reappear/06_-_Chance_Luck_Errors_in_Nature_Fate_Destruction_As_a_Finale>.
